# Envisioning the future for families running away from war: Challenges and resources of Ukrainian parents in Italy

**DOI:** 10.3389/fpsyg.2023.1122264

**Published:** 2023-03-15

**Authors:** Patrizio Paoletti, Giulia Federica Perasso, Carmela Lillo, Grazia Serantoni, Alessandro Maculan, Francesca Vianello, Tania Di Giuseppe

**Affiliations:** ^1^Fondazione Patrizio Paoletti, Assisi, Italy; ^2^Research Institute for Neuroscience Education and Didactics, Fondazione Patrizio Paoletti, Assisi, Italy; ^3^Department of Philosophy, Sociology, Pedagogy, and Applied Psychology, University of Padua, Padua, Italy

**Keywords:** refugees, neuropsychopedagogical training, parents, resilience, positive resources, Ukraine, asylum seekers, war

## Abstract

Since February 2022, 7.8 million people have left Ukraine. In total, 80% are women and children. The present quali-quantitative study is the first in Italy to (i) describe the adaptation challenges and the resources of refugee parents and, indirectly, of their children and (ii) investigate the impact of neuropsychopedagogical training on their wellbeing. The sample includes *N* = 15 Ukrainian parents (80% mothers, mean age = 34 years) who arrived in Italy in March and April 2022. The parents participated in neuropsychopedagogical training within the program Envisioning the Future (EF): the 10 Keys to Resilience. Before the training, participants completed an *ad hoc* checklist to detect adjustment difficulties. After the training, they responded to a three-item post-training questionnaire on the course and to a semi-structured interview deepening adaptation problems, personal resources, and the neuropsychopedagogical training effects. Participants report that since they departed from Ukraine, they have experienced sleep, mood, and concentration problems, and specific fears, which they also observed in their children. They report self-efficacy, self-esteem, social support, spirituality, and common humanity as their principal resources. As effects of the training, they report an increased sense of security, quality of sleep, and more frequent positive thoughts. The interviews also reveal a 3-fold positive effect of the training (e.g., behavioral, emotional-relational, and cognitive-narrative).

## 1. Introduction

According to the United Nation Office for the Coordination of Humanitarian Affairs (2022) report—updated on 23 November 2022—since 24 February 2022, more than 7.84 million Ukrainians (a quarter of the country's total population) have fled their homes in the face of the Russian-Ukrainian conflict and sought refuge in Europe. An estimated 40% are children (Save the Children, 2022). According to the scientific literature, refugee children and adolescents may develop post-traumatic stress disorder, anxiety, and depression symptoms, mainly related to the protracted sense of threat to their safety (Bronstein and Montgomery, 2011). As with natural disasters and other widespread socio-political conflicts around the world (Fazel and Stein, 2002; Taylor and Sidhu, 2012; Cai et al., 2022; Tandon et al., 2022), the conflict between Russia and Ukraine can threaten the mental health of refugee children and adolescents.

In families fleeing war, special attention must also be paid to parents. Higher levels of anxiety and psychological distress in parents predict internalizing and externalizing behaviors in their children, as shown in a study of refugees from Eritrea (Betancourt et al., 2012a) and Colombia (Flink et al., 2013). A study by Hosin et al. (2006), on refugees from Iraq, reported that children of refugee parents may also show lower adaptation skills. Conversely, having parents who can offer care, listening, and respect is a predictor of lower levels of anxiety in refugee children from Chechnya (Betancourt et al., 2012b). Similarly, a study of Syrian refugee children revealed that a family climate that encourages emotional expression decreases the risk of developing PTSD symptoms (Khamis, 2019).

It has been underlined by ethnographic research that resilience is a key resource for parents who are going through alone such a severe situation (Lenette et al., 2013). In such a population, resilience should be fostered through specific educational programs (Paoletti et al., 2022a). These activities allow the individuals to be fortified by trauma and adversity, turning challenging events into opportunities to improve oneself in the present and future (Grotberg, 1995), including the experience of parenting. Specifically, in response to humanitarian crises, programs that integrate combined complementary approaches (Kalmanowitz and Ho, 2016) can facilitate improvements in refugees' quality of life (Chung and Hunt, 2016) by reducing negative symptoms of stress, intrusive thoughts, and sleep disturbances (Rees et al., 2014) and helping them to view adversity situations with a more positive mindset (Goehring, 2018).

Refugee parents, who are subjected to high levels of stress, may be compromised in their ability to care for their children. Therefore, their children's health must also be implemented and protected by, in turn, promoting the health of their caregivers (Riber, 2017; Buchmüller et al., 2018; Scharpf et al., 2021) and implementing their overall wellbeing, as individuals and as parents, under adversity.

In consideration of the premises highlighted in the literature, it is a priority to monitor the health and coping strategies of refugee parents in the host country and implement interventions that are helpful to increase the level of wellbeing in these populations.

## 2. Study aims

The aims of this research note were two-fold: (i) to describe both the adaptation challenges faced by Ukrainian parents, and indirectly by their children, and the main personal resources they have put in place since their arrival in the host country; (ii) to investigate the impact of neuropsychopedagogical training on the wellbeing of Ukrainian parents and the indirect impact on their children.

## 3. Materials and methods

### 3.1. Procedure

Neuropsychopedagogical training based on the program “The 10 Keys to Resilience” (Paoletti et al., 2022b) took place in the context of an educational campus targeting Ukrainian families fleeing war, held from 13 June to 22 July 2022, organized by Fondazione Patrizio Paoletti (FPP) with the support of Assisi International School (AIS). The program has been applied in several emergency settings, in Italy, as part of the multi-year Envisioning the Future (EF) project (Di Giuseppe et al., 2022, 2023; Maculan et al., 2022; Paoletti et al., 2022b). The goal of EF is to provide individuals and communities with an educational pathway to strengthen the resilience and resources of people engaged in the care of children and adolescents. The educational intervention and related research were conducted with the approval of the Ethics Committee of the University of Padua (Protocol No. 0003662). At the campus, the educational materials used were translated into Ukrainian.

The theoretical-practical framework of the program “The 10 Keys to Resilience” is based on the Sphere Model of Consciousness (Paoletti and Ben-Soussan, 2019; Pintimalli et al., 2020) and Pedagogy for the Third Millennium (Paoletti, 2008), developed by the FPP interdisciplinary team, which includes guidance based on neuropsychopedagogical knowledge and techniques for coping with stress and difficulties. The program has been successfully implemented among educators in the juvenile penal circuit (Paoletti et al., 2022c, 2023), among inmates during the COVID-19 pandemic (Di Giuseppe et al., 2022), and among earthquake survivor communities (Di Giuseppe et al., 2023). The “10 keys to resilience” are as follows: (1) restart from what you can control and make small decisions; (2) identify an attainable, challenging, and measurable goal; (3) several times a day become aware of your posture; (4) be inspired by the others stories; (5) ask yourself what is really important; (6) cultivate gratitude; (7) experience the other as a resource, cultivate and expand your social network; (8) cultivate curiosity; (9) practice a few minutes of silence; (10) embrace and transform: before going to sleep, generate today your own tomorrow (for more information, see Paoletti et al., 2022a,b).

The program provided for Ukrainian parents to benefit from the blended training (e.g., online and in-person activities) “The 10 Keys to Resilience,” through 14 meetings (average duration of 2.5 h), with simultaneous translation and training materials provided in both Italian and Ukrainian. The presence of a cultural mediator of Ukrainian nationality was provided both in the design and creation phase of the content and in its fruition, ensuring not only translation but also a relational link between teachers and participants.

The program included two intervention focuses: self-focused, for the development of parental resilience and positive resources, and child-focused, for the strengthening of parenting skills during adversity.

### 3.2. Participants

In total, 15 Ukrainian parents (mean age = 34 years, 80% mothers), who were subject to informed consent, took part in the study. They arrived in Italy (Umbria), with their children, through the support of Ukrainian relatives, friends, and acquaintances, already residing in Italy. The latter created on their own initiative communities of welcome and support for their refugee compatriots (providing them with accommodation, food, and the possibility of temporary job placement). The participants, welcomed by these spontaneous communities, reported leaving Ukraine 20% in April and 80% in March 2022. Most of the refugee participants were from the city of Kyiv (46%), with the remainder coming from Ivano-Frankivsk (27%), Kharkiv (7%), Irpin (7%), Smila (7%), and Dnipro (6%). A total of 53% were mothers who reported being in Italy without the child's other parent.

### 3.3. Measures

The measures were created *ad hoc* following the principles of psychology research methods (Kazdin, 2013). Pre-training, participants completed an *ad hoc* self-administered checklist on their own and their children's biopsychosocial health status. The checklist allows them to report dichotomously (e.g., by answering yes or no to each item) on the occurrence, since leaving Ukraine, of problems related to adaptation (e.g., lack of attention, negative mood, specific fears, irritability, physical symptoms, appetite-related difficulties, sleep-related difficulties, and, in children, also difficulties in relating to peers and playing).

Post-training, an *ad hoc* questionnaire was also administered: it consisted of three items related to the program experience on “The 10 Keys to Resilience,” which investigated dichotomously (with yes or no answer options) improvements with respect to the participant's sleep quality, increase in positive thoughts, and increased perception of safety.

In addition, post-training, participants also responded to individual semi-structured interviews, conducted by a psychologist, about issues of adaptation, major difficulties since arrival in Italy, and the impact of the training. The interviews were conducted in the presence of a cultural mediator of Ukrainian nationality, in line with the literature that posits the mediator as an active participant in the interview and not just a translator (Raga et al., 2020), capable of creating a link between the interviewer and the interviewee.

### 3.4. Data analysis

To investigate the defined aims, two studies were implemented. The first one aimed at a quali-quantitative analysis of the main personal resources and adjustment challenges of Ukrainian parents, and—indirectly—the difficulties of their children. For this purpose, descriptive statistics were performed on the data from the *ad hoc* self-administered checklist and bottom-up text analysis on the verbatim transcripts of the semi-structured interviews, with subsequent categorization.

The second study aimed at a quali-quantitative assessment of the impact of “The 10 Keys to Resilience” training on Ukrainian parents. For this purpose, descriptive statistics were performed on the responses to *ad hoc* post-training dichotomous items. To understand the effect of the training, according to the two lines of intervention (self-focused and child-focused), the transcripts of the interviews were analyzed in a bottom-up approach, extracting the main categories.

For the categories that emerged in the two studies, the analysis of the interviews involved the statistical calculation of the inter-rater agreement between two different evaluators using Cohen's K. The evaluators were to score the relevance of the extracts related to each category on a scale from 0 = not relevant to 3 = totally relevant.

## 4. Results

### 4.1. Study 1: Adjustment problems and personal resources

Most parents report that in recent months, they are experiencing sleep-related difficulties (80%), negative mood (70%), lack of attention and concentration difficulties (60%), specific fears (60%), and irritability (63%) (Table 1). Similarly, they report that their children are experiencing mostly problems related to attention (60%), mood (60%), irritability (60%), and specific fears (40%) (Table 1).

**Table 1:** Post-traumatic symptoms in Ukrainian parents and children.

	**Sleep**	**Appetite**	**Relationship with peers**	**Physical symptoms**	**Irritability**	**Specific fears**	**Negative mood**	**Attention**
Parents	80%	40%	0%	20%	63%	60%	70%	65%
Children	30%	30%	30%	0%	60%	40%	60%	60%

Parents' main adjustment problems, as revealed by the analysis of the semi-structured interviews (Table 2), appeared to be related to the language and bureaucratic barrier (40%), prolonged permanence in Italy (13%), job loss (13%), and family issues (6%). Difficulties in caring for children (20%) and, for the latter, drastic changes in their routines (6%) also emerged (Table 2). At the same time, the main resources deployed by parents appear to be self-efficacy (26%), perceived social support (66%), spirituality (6%), common humanity (13%), and self-esteem (13%). The inter-rater agreement appears to be high (Cohen's K = 0.88).

**Table 2:** Excerpts of the main areas related to adjustment problems and personal resources of Ukrainian parents (*N* = 15).

**Main area: Adjustment problems**	**Excerpts**
Language and bureaucratic barriers	L200193: the difficulties are first of all of the fact that it's another country, it's other rules, other language, other customs, it's all different ... the difficulties initially were with the documents and with the house, life, where to live, space, moving, all the organizational issues
Prolonged permanence in Italy	L200193: First of all the understanding that I am here and I have a shock, a shock because I thought it would be for two months, that I would wait here for two months and afterwards I would go back to my home. Now I understand, I realize that I will stay here
Job loss	I199232: My whole life, all my work is there, I'm an artist, a freelancer all my life, since I was a little girl I've always been earning as an artist, and so all the connections, my network is there ... It would be nice to solve the work issue now to be able to engage in ordinary normal work as well
Family issues	M1999229: the difficulties I can say are right now within the family, for example with my husband because I would like to come back but he does not agree, and this is also my inner conflict
Difficulties in caring for children	M1999229: With three children certainly it is an economic issue, you have to earn money to get by because they have needs and wants
Drastic changes in children routines	N198329: The second difficulty, or challenge, if we can call it that, is our regime that has been disrupted, destroyed completely, everything that I built in the first four years in Ukraine, cartons, sweets, telephone, all those rules that we built to give us a regime, today are gone. in the beginning we were all under stress and it was difficult because we were all in the news trying to figure out what was going on, or we were documenting what we have to do here, how things work here, what are the procedures, and so I was busy with that, now that a little bit this part is clearer I'm getting her back to her routine, her rules, her regimes. Plus I was telling her that we are going on vacation, eating gelato in Italy, so also for her it was a time without rules, without obligations, without a routine, now instead we are resuming classes with the teacher we had in Ukraine but online currently, and then there are other things that are coming back into her life
**Main area: Personal resources**	**Excerpts**
Self-efficacy	E198310: self-efficacy, being open, don't close yourself off. Finding, doing what you love, what you enjoy in order to access your resourceful state, to then be able to do what you need to do
Self-esteem	E198310: since we moved, already after a month or so since we moved out of the danger territory, so out of the life danger, I realized that I had an inner prohibition to happiness, and so I was not allowing myself joy, happiness, then then I realized for example the things that are very important for women: makeup, looking good, even that I felt guilty about, and the first time for example I wore makeup when we went to do the documents, the pictures for the documents, I realized how important it is to like yourself in the mirror, to have a look that you like about yourself, it supports you, it sustains you, it gives you that strength to face things. Then I realized that my being sad in the moment, it will not be able to support, help people, children who are dying, these are terrible things that happen, but not with my sadness I will be able to be supportive of these situations
Social support	L200193: they welcomed us very well, certainly, I can say that they welcomed us not as people belonging to a nationality because I really feel that this is the place where there is no distinction between country of belonging, they welcomed us as people, I say what kind of person are you ... It was a very good moment when people here started to help us. When you receive, when you see people helping, you feel like going on and continuing your journey, and giving back to people
Spirituality	I199232: Faith and trust that everything that happens to me is for my best, faith is in the Almighty, in God
Common humanity	N194998: I think it's essential that people remain human, because to think that in the 21st century not only so many friends but also so many children die is unbearable, unacceptable today, so I think you have to have faith ... hope is certainly love for humanity

### 4.2. Study 2: The impact of the “10 Keys to Resilience” program

According to the statistical-descriptive results, on the post-training questionnaire, Ukrainian parents report that they perceived improved sleep (80%), increased confidence (86%), and more frequent positive thoughts (86%) after the training on the “10 Keys to Resilience.” The impact of the training was further explored by text analysis of the interview excerpts (Table 3).

**Table 3:** Excerpts from the interviews with Ukrainian parents participating in the intervention (*N* = 15).

**The impact of the “10 keys to resilience”: Main areas**	**Self-focused excerpts**	**Child-focused excerpts**
Behavioral area (Keys 1, 3)	M198669: the first one that helped me was key 1, the one that says “don't try to understand or give answers to everything around you, but try to break it down into small steps and do what you can do now.”	E198310: The unraveling, the biggest discovery for me was the example of not telling the children what to do, but showing them because they imitate us, they copy us ... So before I was very much in the position of “do this, do this,” instead now it's “let's do together, let's thank together,” or I realized that it's important, when I do something, to involve them in what I do, and so we do a planning of the day together ... I realized that maybe I lacked time in the last life, instead now I have more time to participate in their things, to participate in their life
	N198329: Do what depends on you right now. That's the key that I tried to implement when we arrived, that's what I asked myself and said when we arrived, I realized that I have to take care of my family, I have to take care of, and so I have to get the resources, the food, and so that's what I need to have the strength to deal with what I have to do	Y198380: In the relationship with the daughters, I realize that it is useful to teach them, to instruct them to things, and what I have seen is that it is very interesting and useful to make them see, to teach them through their own example, it allows them to copy my useful behaviors intentionally
Emotional-relational area (Keys 4, 5,6,7)	E198310: now in the situation I found myself in, the key that is inhabiting me is that of “be thankful”. I understood it in a new way, it's not saying thank you out of kindness, out of courtesy, as we used to do, it's experiencing that emotion of gratitude, and when you pass it through you the things that come to you are greater	I199232: (with my daughter) we have always had a good relationship because she is my mirror, so when I am better this thing is also reflected in her, certainly, so certainly it has helped me because if I grow, in her this thing is reflected ...
	A198535: I always thought that you have to take the good from others, the beautiful things, but there we said that even if the experience, the people around you are not beautiful, beautiful, it is still an experience, and it is always a resource	M198669: another very important key that we are using is to build the social network, widen the social network, in fact I'm talking a lot with my daughter, I'm telling her that as we ended up today in Italy, tomorrow we could end up in another country, in new circumstances, in new conditions and even there she should not be afraid to build new relationships because those are the ones that then allow you to both solve problems, and then to have people around you with whom to share a life path
Cognitive-narrative area (Keys 2, 8, 9, 10)	M1999229: first of all be patient, certainly remember that nothing is permanent, even this moment, whatever it is, will pass, everything will pass, and then remember that you can achieve, you can arrive, give yourself goals and tell yourself that you can achieve them because this moment is not static, it is not stable, it will pass, it is not permanent	I199232: I have seen that it has improved with her (my daughter), this thing of seeing from multiple points of view, a negative thing from a positive point of view
	N194998: this minute (of silence) is not just a minute, it's a healing minute, it heals you in this minute, not only because you rest, you can rest in the sense of not thinking about it, or you can dwell on something that is really important to you. It rests you, it heals your body, it rests your emotions, it orders your thoughts, then I would recommend it to everybody, to today's moms, to all people	E198310: definitely now is to help the child to build, to define his goal and detail it in the little steps that can help him to reach it, and to remind him daily, involving him, not telling him “do do do,” which was my position before, but to do it together and to be part of that, and it's also my problem to detail, I see the goal and I want everything and right away, and so detailing, defining the little steps is important, I'm doing that. And then another important thing I started to do is to, after each little step, to celebrate, to emphasize, to say “bravo, bravo, you did well,” to support

**Figure 1:**
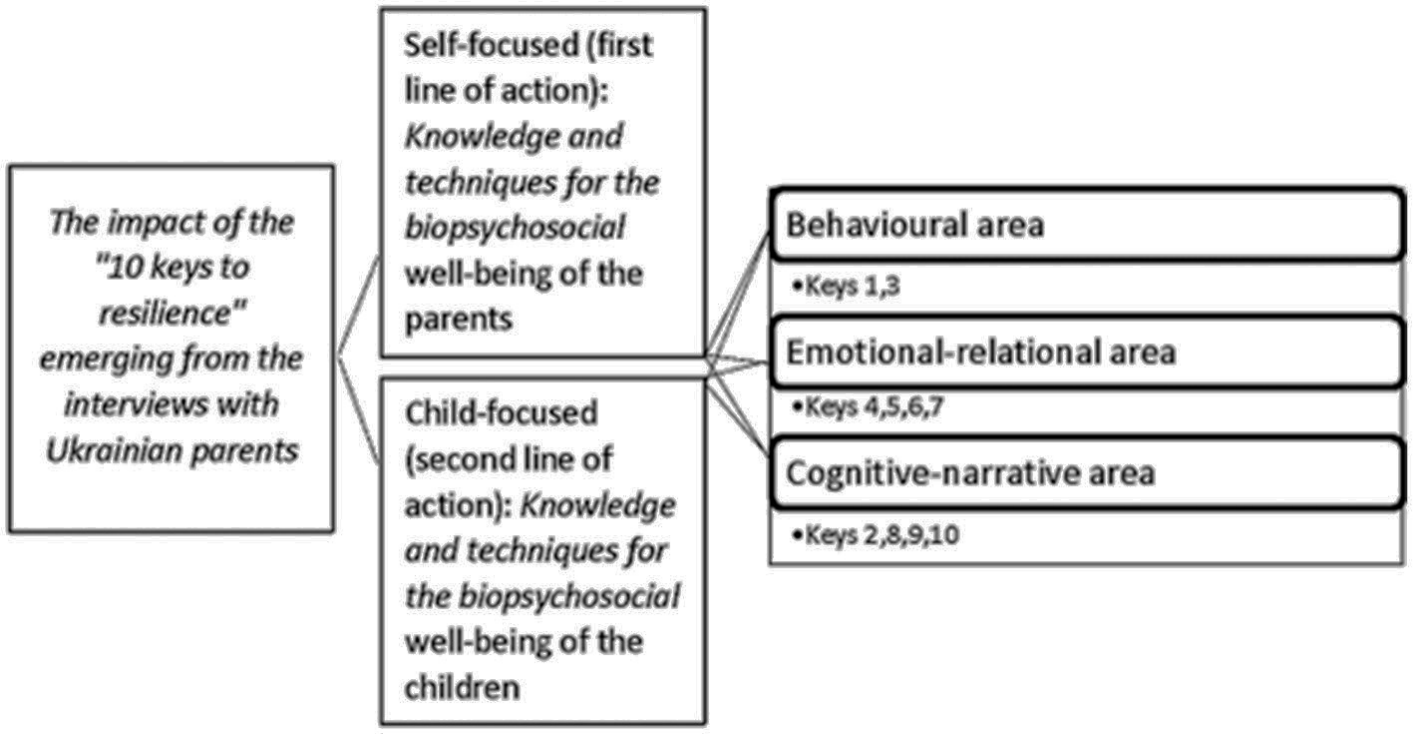
Impact of the “10 Keys to Resilience” emerging from the interviews with Ukrainian parents.

Ukrainian parents reported that the “The 10 Keys to Resilience” training provided them with theoretical-practical instruments, to be used for their own (self-focused line of intervention) and children's (child-focused line of intervention) wellbeing, concerning three specific areas (Figure 1): (i) behavioral (keys 1 and 3); (ii) emotional-relational (keys 4, 5, 6, and 7); (iii) cognitive-narrative (keys 2, 8, 9, and 10). The inter-rater agreement is found to be high (Cohen's K = 0.88).

## 5. Discussion

### 5.1. Ukrainian refugee parents in Italy: Principal features

#### 5.1.1. Adaptation challenges

Study 1 results show that Ukrainian refugee parents experience mood-related problems and irritability. This finding is in line with recent studies investigating both migration flows caused by wars and the COVID-19 pandemic reactions, showing the prevalence of emotional dysregulation, anxiety, and post-traumatic stress in individuals, across different nations and socio-political conditions (Henkelmann et al., 2020; Turliuc and Candel, 2022). In addition, they report sleep problems related to both migration stress and broader post-traumatic symptomatology (Richter et al., 2020). These elements may also cause the lack of concentration that the participants' report (Kaplan, 2009) and the experience of specific fears and phobias (Thapa et al., 2003; Betancourt et al., 2017).

Regarding the issues parents report in their children, the literature highlights a higher incidence of PTSD among refugee children and adolescents than among adults (Henkelmann et al., 2020): a statistical comparison was not performed in this study, but issues of irritability and negative mood in children are defined as salient by their parents. Similarly, adults report a lack of concentration in children that could be due to the effects of trauma, with a possible negative impact on cognitive development and learning abilities (Beers and De Bellis, 2002; Kaplan et al., 2016). Finally, specific phobias and fears may emerge in the child subjected to traumatic situations (e.g., detachment from one of the parents, from one's routine, from one's home, and from one's parental and friendship circle), as witnessed by the parents, which may negatively influence development (Javanbakht et al., 2021).

The adaptation difficulties that emerged from the semi-structured interviews concern multiple topics: forced migration, language and bureaucratic difficulties, sense of uncertainty, unemployment, and taking care of the children. First, the dramatic circumstances in the participants' home country lead them to a forced migration where the destination country was not chosen (Becker, 2022). Language difficulties amplify the sense of alienation in the new context, and language constitutes a barrier to the fulfillment of bureaucratic matters (Gerlach and Ryndzak, 2022), undermining the individual's sense of autonomy as well as their perceived ability to manage their family. Other sources of concern include the sense of uncertainty about the future provoked by the ongoing war in Ukraine (Mohamed and Thomas, 2017; Becker, 2022) and the unemployment status which can predict depression among refugees (Beiser et al., 1993). Adaptation difficulties are also reported in the management of children: refugee parents are worried about their mental and physical health status (Javanbakht, 2022), given the disruption of their before-war routines.

#### 5.1.2. Adaptive resources

In Study 1, five adaptive resources emerged from the semi-structured interviews of Ukrainian refugee parents: self-efficacy, social support, spirituality, sense of common humanity, and self-esteem. Being a refugee could decrease individual empowerment (Bowie et al., 2017). However, among the resources deployed by parents, self-efficacy appears to strengthen coping by supporting them in raising children (Slagt et al., 2012; Vintilă and Istrat, 2014). More specifically, self-efficacy predicts greater relational involvement (Shumow and Lomax, 2002) and greater support and ability to maintain general wellbeing (i.e., their own and the children) (Deković et al., 2010; Goian, 2014; Kalaitzaki et al., 2022), factors associated with positive adaptation (Meunier and Roskam, 2009).

Another key resource is the perceived social support both externally and within the refugee community. This finding is in line with previous research highlighting the importance of social support in decreasing the risk of PTSD and increasing adaptation among Sudanese refugees in Australia (Schweitzer et al., 2006) and Afghans in Canada (Ahmad et al., 2020). Specifically, a study by Stewart et al. (2017), on refugee parents from Sudan and Zimbabwe in Canada, reports that social support is perceived as higher if it comes both from the refugee community and from professionals who are sensitive to cultural differences issues. An additional resource mentioned by Ukrainian parents is spirituality, consistent with evidence on spirituality as a coping resource associated with greater hope and optimism in refugees (Ai et al., 2003), especially among refugee parents (El-Khani et al., 2017).

Participants refer to perceive a sense of closeness with other human beings. This data could be interpreted in light of Neff (2011) “Common Humanity,” a subdimension of self-compassion, which describes the sense of feeling as a part of humanity. Finally, perceiving intact self-esteem is mentioned by the refugee parents as a basic element for psychological wellbeing (Neff, 2011).

#### 5.1.3. The impact of the neuropsychopedagogical training

In Study 2, participants responses to the post-training questionnaire reveal an improvement in sleep after the training. In this sense, the intervention may be a protective factor against the chronicization of sleep disorders, which can remain in refugees long-term after the traumatic events, with negative consequences for individual health (Basishvili et al., 2012; Richter et al., 2020). The responses to the questionnaire, “The 10 Keys to Resilience” within the EF program, appear to increase the frequency of positive thoughts, in line with the outcomes of positive psychology interventions on refugees (Kubitary and Alsaleh, 2018; Foka et al., 2021), to facilitate post-traumatic growth through hope (Umer and Elliot, 2021). Parents report that EF increased their sense of security, comforting them about their newfound safety but also about the possibility of constituting, in attachment terms, a secure base for themselves and their children (Dalgaard et al., 2016). Moreover, by helping parents to focus on their own positive resources, the intervention could have played an important role to prevent the feeling of “subjective incompetence” in stressful situations, described in the literature as demoralization (Costanza et al., 2022a).

Extracts from the participants' interviews describe the positive impact of the training on the behavioral, emotional-relational, and cognitive-narrative areas, highlighting the importance of providing the Ukrainian population with psychological support tools (Costanza et al., 2022b).

As regard the behavioral area, the training encouraged participants to perceive themselves as active subjects, who can exert control over their environment. Having a greater sense of control promotes safety and a reduction in anxiety and negative emotions (Gallagher et al., 2014). Participants refer an increase in wellbeing and coping through small actions. This data also reflects the importance for parents of being an example for their children and engaging them in practical actions to build together new reassuring routines, able to mitigate uncertainty. As reported in the literature, in times of severe crisis, being able to decide which simple and regular activities to be engaged in is fundamental. This allows persons to reinforce the perception of a safe and stable surrounding (Nelson and Shankman, 2011; Bentley et al., 2013), as experienced by the refugee parents through EF.

Regarding the emotional-relational area, refugee parents felt gratitude, an emotional state associated with wellbeing, in a relationship mediated by perceived social support (Lin, 2016). Social support is a key resource for refugees' mental health and resilience (Schweitzer et al., 2006; Stewart et al., 2017; Sim et al., 2019; Ahmad et al., 2020) and a positive coping strategy (El-Khani et al., 2017). Perceiving higher social support may lead individuals to want to show reciprocity and be helpful to others in turn (Schmitt, 2021) as emerged in the interviews.

Finally, the intervention seems to have had an impact on the cognitive-narrative area. The use of cognitive strategies, such as problem-solving, planning, and positive reappraisal, helps cope with stressors (Feder et al., 2009) and makes it possible to experience a minor impact of war-related trauma and life changes related to refugee status (Fino et al., 2020). The practice of silence-based meditation (Paoletti, 2018), suggested in the program, also impacts the cognitive-narrative area, and it is described by the participants as a powerful tool for activating a re-narration of experiences through self-talk, in the form of a “silent narrative” (Morin, 2009; Kross et al., 2014). The practice of silence-based meditation is mentioned as a healing experience, in line with studies on the therapeutic effect of meditative practices on refugees (Hinton et al., 2013; Kalmanowitz and Ho, 2016), and as a restful experience, promoting mental functioning and reducing alertness and stress symptoms (Rees et al., 2014).

## 6. Limitations and future directions

The first limitation of the present research is the small sample size, which does not allow result generalization. However, sample size standards in qualitative research are linked to historical and cultural factors related to the target sample (Marshall et al., 2013). The second limitation is the use of the semi-structured interview, even in the presence of a cultural mediator, that could generate social desirability bias in refugee samples (Da Silva Rebelo et al., 2022).

Notwithstanding these limitations, this is the first study in Italy to investigate the adaptation problems and challenges of Ukrainian parents and at the same time the effects of neuropsychopedagogical training on their wellbeing. The study lays the foundation for subsequent investigations into the biopsychosocial health of Ukrainian refugees, especially concerning the experience of parenting and interventions aimed at supporting them. It is suggested for future studies in the field to implement a multidisciplinary approach that integrates clinical and socio-educational interventions, which should be longitudinally evaluated.

## 7. Conclusion

The present findings describe the complexity of the experience of Ukrainian refugee parents, who arrived in Italy between March and April 2022. The quantitative analysis highlights the negative impact of refugee status on the wellbeing of Ukrainian parents. However, attention is also paid to the positive resources emerging from the qualitative analysis of their interviews. It is precisely this aspect that the EF training sought to enhance to support Ukrainian refugee parents. The training allowed the refugee parents not only to improve their coping strategies but also to renew their awareness of their role as a parent, guiding their children in times of complexity. The interviews' extracts highlight their role, not only as protectors and caregivers of their children but also as active educators promoting their mental and emotional wellbeing.

The research is the first neuropsychopedagogical experience, in Italy, to apply a specific theoretical and methodological framework to safeguard the wellbeing of Ukrainian refugees, educating primary educators (e.g., parents) in the functioning of the resilient mind and transmitting usable long-term techniques (e.g., the practice of silence). The interdisciplinary approach recalls the importance of monitoring the wellbeing of refugees—adults and, indirectly, children. The study highlights the timeliness of intervening to transform the traumatic experience into a source of resources to face the present and future.

## Data availability statement

The raw data supporting the conclusions of this article will be made available by the authors, without undue reservation.

## Ethics statement

The studies involving human participants were reviewed and approved by Ethics Committee of the University of Padua (Protocol No. 0003662). The patients/participants provided their written informed consent to participate in this study.

## Author contributions

PP, TD, CL, and GP contributed to the design and implementation of the research. GP, CL, and GS analyzed quantitative and qualitative data. GS, AM, and FV helped with the references. PP structured the theoretical framework as the developer of the Sphere Model of Consciousness and Pedagogy for the Third Millennium. GP, LC, and TD drafted the manuscript. TD integrated and coordinated the study. All authors provided substantial contributions to the study, critically revised the manuscript, approved this version, and agreed to be accountable for all aspects of this study and its integrity.
